# Diagnostic Performance of Artificial Intelligence Corrected OCT Measurements in Highly Myopic Eyes with Glaucoma

**DOI:** 10.3390/jcm15114320

**Published:** 2026-06-03

**Authors:** Patricia Robles Amor, Alfonso Antón López, Susana Duch Tuesta, Javier Moreno Montañés, Francisco José Muñoz Negrete, Ignacio Rodríguez Uña, Laura Morales Fernández, Federico Sáenz Francés, Julián García Feijoó, José María Martínez de la Casa, on behalf of GlaucoAI-Spain

**Affiliations:** 1 Hospital Clínico San Carlos, Instituto de Investigación Sanitaria del Hospital Clínico San Carlos (IdISSC), Universidad Complutense de Madrid, 28040 Madrid, Spain; 2 Institut Català de Retina, Universitat Internacional de Catalunya, 08022 Barcelona, Spain; 3 Verte-ICO Oftalmología, 08006 Barcelona, Spain; 4 Clínica Universidad de Navarra, 31008 Pamplona, Spain; 5 Hospital Universitario Ramón y Cajal, Instituto Ramón y Cajal de Investigación Sanitaria (IRYCIS), Universidad de Alcalá, 28034 Madrid, Spain; 6 Instituto Oftalmológico Fernández-Vega, Universidad de Oviedo, 33012 Oviedo, Spain

**Keywords:** high myopia, glaucoma, deep learning, artificial intelligence, optical coherence tomography

## Abstract

**Objectives:** This study aimed to evaluate the diagnostic performance of peripapillary retinal nerve fiber layer (RNFL) thickness measurements corrected by artificial intelligence (AI) compared to original uncorrected values for glaucoma detection in highly myopic patients. **Methods:** This cross-sectional diagnostic accuracy study included 57 eyes from highly myopic patients (31 with glaucoma, 26 without glaucoma). Peripapillary RNFL parameters were obtained using Spectralis optical coherence tomography (OCT). A deep learning algorithm (MGU-Net) was employed to automatically segment retinal layers and compensate for scan tilt in elongated eyes, producing AI-corrected measurements. RNFL thickness values were extracted for six sectoral parameters (ST, SN, N, IN, T, IT) and global. Diagnostic performance was assessed using area under the ROC curve (AUC) and compared between corrected and uncorrected values. Multivariable logistic regression models were also developed using stepwise selection. **Results:** AI-corrected values were significantly lower than original measurements in all sectors (*p* < 0.001), with mean differences ranging from 15 to 35 µm. In glaucomatous eyes, significant thinning was observed in the global (*p* = 0.049) and inferior nasal (IN) sector (*p* = 0.037) among corrected values. The highest AUCs were found in IN (0.69), IT (0.67), and global (0.66) for corrected values, and in IT (0.63), T (0.59), and global (0.63) for uncorrected data. A model combining ST, T, and IT AI-corrected values achieved an AUC of 0.79. **Conclusions:** AI-corrected RNFL thickness measurements improve consistency and enhance diagnostic performance in highly myopic glaucoma patients. Correction algorithms may reduce false positives and help reveal glaucomatous damage otherwise obscured by myopic anatomical changes.

## 1. Introduction

Glaucoma is a leading cause of irreversible blindness globally, characterized by progressive optic nerve damage and visual field loss. High myopia, defined as refractive error greater than −6 diopters or axial length exceeding 26 mm, significantly increases the complexity of glaucoma diagnosis due to overlapping structural changes.

Diagnosing glaucoma in highly myopic eyes presents significant challenges when relying on Optical Coherence Tomography (OCT). Myopic eyes often exhibit anatomical alterations such as optic disc tilt, peripapillary atrophy, and increased axial length, which can lead to thinning of the retinal nerve fiber layer (RNFL) in the absence of disease. These structural changes can mimic glaucomatous damage and result in a high rate of false-positive diagnoses when compared to standard normative databases [1]. Moreover, studies have shown that increasing axial length is directly correlated with decreased RNFL thickness, independent of glaucomatous pathology [1,2]. These findings underscore the need to account for axial elongation, magnification effects, and age-related progression rates when interpreting OCT data in myopic patients. Given these limitations, there is growing interest in alternative OCT parameters such as Bruch’s membrane opening-minimum rim width (BMO-MRW) and vascular density measures, which may provide improved specificity in this subgroup [3,4].

OCT is a fundamental imaging modality in glaucoma diagnosis, offering quantitative and reproducible measurements of key structural parameters such as peripapillary RNFL thickness, ganglion cell–inner plexiform layer (GCIPL) thickness, and neuroretinal rim area. These parameters enable the early detection and monitoring of glaucomatous damage in routine clinical settings. However, in highly myopic eyes, the accuracy of OCT is compromised due to anatomical variations such as tilted optic discs, peripapillary atrophy, and posterior staphyloma, which interfere with image segmentation and interpretation. RNFL thickness measurements, while commonly used, tend to decrease with increasing axial length and are subject to ocular magnification effects, often resulting in false-positive diagnoses [2,5]. In contrast, macular GCIPL thickness has demonstrated superior diagnostic performance in some studies, with reported AUROC values as high as 0.951, surpassing RNFL in certain high myopia populations [6]. Similarly, alternative optic nerve head parameters such as three-dimensional neuroretinal rim thickness (3D-NRR) and BMO-MRW have shown promise in reducing false positives [1,3]. These findings underscore the need for tailored OCT interpretation in myopic patients, including the use of myopia-specific normative databases and a combined parameter approach to enhance diagnostic accuracy [7]. Recent advances in artificial intelligence (AI) have opened new avenues for improving glaucoma diagnosis, particularly in complex cases such as high myopia where traditional OCT interpretation is often unreliable. Deep learning algorithms have demonstrated high diagnostic accuracy when applied to OCT imaging, with pooled sensitivities ranging from 0.91 to 0.98 and specificities from 0.90 to 0.98 across multiple models and studies [8,9]. These approaches often outperform conventional OCT interpretation, which lacks standardized diagnostic thresholds and is prone to human error, especially in anatomically atypical eyes. AI-corrected OCT may thus reduce false positives and improve reliability in myopic populations by compensating for segmentation errors and anatomical distortions. In this study, we aim to compare the diagnostic performance of original versus AI-corrected peripapillary RNFL thickness measurements for glaucoma detection in highly myopic eyes. We hypothesize that AI-corrected values will maintain or improve diagnostic accuracy while providing more anatomically reliable assessments in this challenging clinical subgroup.

## 2. Materials and Methods

### 2.1. Study Design and Participants

This was a cross-sectional diagnostic accuracy study conducted at Hospital Clínico San Carlos, including 57 highly myopic patients. Participants were divided into two groups: 31 eyes with high myopia and glaucoma, and 26 eyes with high myopia without glaucoma. High myopia was defined as a spherical equivalent of ≤−6.00 diopters and/or axial length ≥ 26 mm. Glaucoma diagnosis was based on characteristic optic nerve head changes and corresponding visual field defects confirmed by standard automated perimetry. Exclusion criteria included ocular diseases other than glaucoma or myopia, previous ocular surgery (except for uncomplicated cataract surgery), and poor-quality OCT scans. In relation to the exclusion criterion of adequate image quality, we emphasize this point, as only images with verified continuity of the retinal layers and correct segmentation were analyzed, in patients without other concomitant ophthalmological pathologies, thereby determining the sample size.

### 2.2. OCT Imaging and Parameters

All participants underwent peripapillary RNFL imaging using Spectralis OCT (Heidelberg Engineering, Heidelberg, Germany). The following RNFL thickness parameters were extracted: superior temporal (ST), superior nasal (SN), nasal (N), inferior nasal (IN), temporal (T), inferior temporal (IT) and global (G). Scans were reviewed for quality, and segmentation errors were corrected when needed using built-in software tools.

### 2.3. AI Correction Algorithm

To correct the retinal layer thickness measurements in highly myopic eyes, a fully automatic two-stage assessment methodology was employed. In the first stage, a deep learning-based segmentation architecture (MGU-Net) was used to identify and delineate retinal layers in circumpapillary OCT scans [10]. This network was fine-tuned using a dataset of manually annotated scans from glaucoma patients to optimize segmentation accuracy for the peripapillary region. The second stage consisted of a measurement and compensation module, which first validated the continuity and integrity of the segmented layers and then adjusted thickness measurements based on the estimated tilt of the scan. The tilt correction was performed by calculating the angular deviation of the retinal curvature using the boundary between the retinal pigment epithelium and choroid as a reference. This compensation accounted for distortions due to axial elongation and provided corrected measurements that more accurately reflect true anatomical thicknesses, thereby enhancing diagnostic reliability in highly myopic eyes.

**Figure 1:**
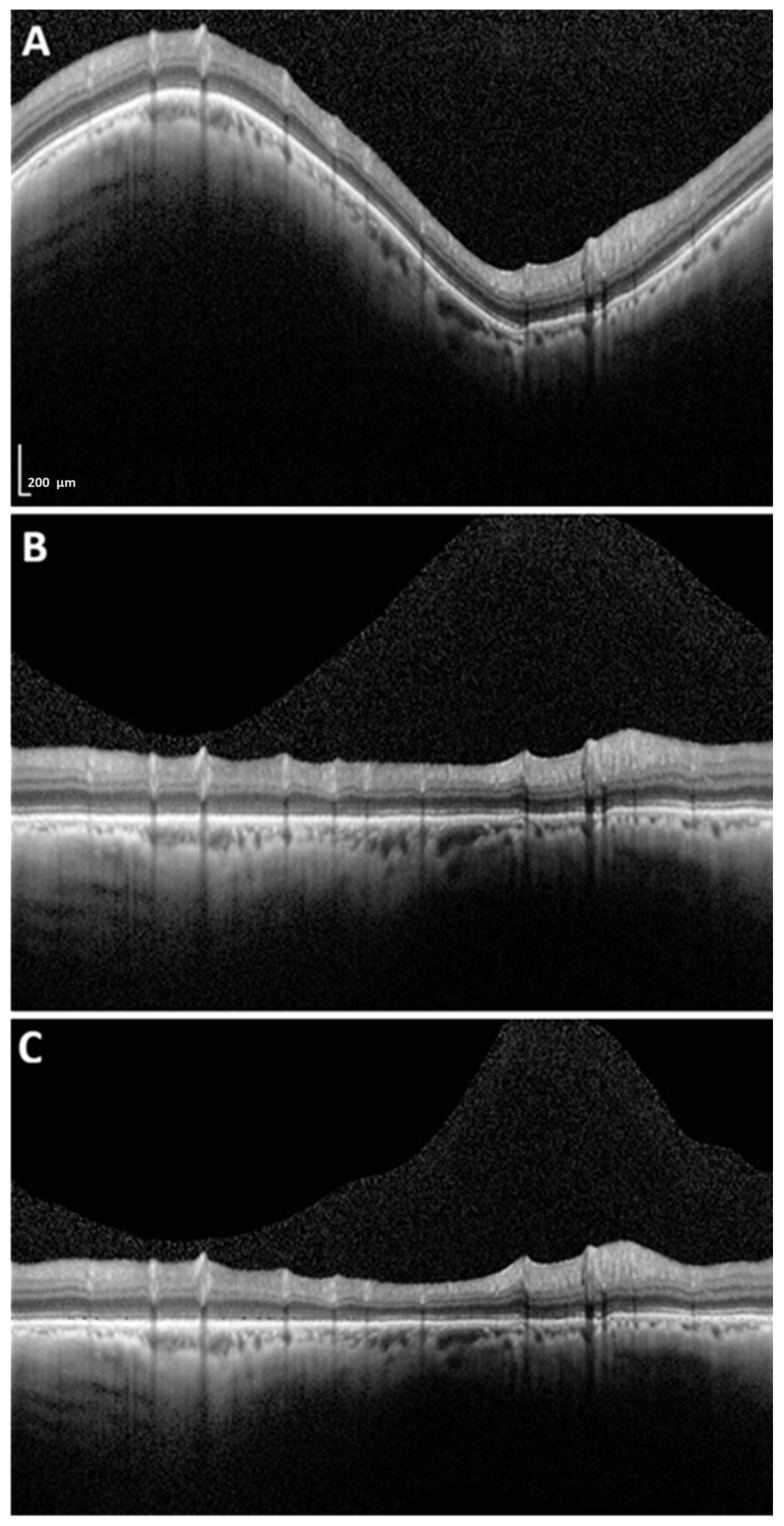
Visual aid to understand the RNFL thickness adjustment process by correcting image tilt. The final processing illustrates the reduction in RNFL thickness provided by the AI-corrected values. (**A**) The scan displays a curved profile caused by the oblique orientation of the optic disc region, which results from the increased axial length associated with high myopia; (**B**) The curvature of the retinal layer profile is corrected; (**C**) The RNFL thickness is adjusted by sector, removing the distortion introduced by the oblique image acquisition. This correction reveals that the actual thickness is thinner than that indicated by the original image.

The following images (Figure 1) are intended to facilitate understanding of the segmentation correction using artificial intelligence; however, it is essential to clarify that they merely represent a visual aid for the reader generated for this sole purpose, with special emphasis on the fact that image modification is not part of the correction algorithm. In the first image (A), the image capture shows a “curved” profile secondary to the obliquity of the optic disc region determined by the increase in axial length in an eye with high myopia. The second image (B) eliminates the curvature of the retinal layer profile, and finally, the third image (C) corrects the retinal nerve fiber layer thickness by sectors, eliminating the distortion secondary to oblique image capture and showing that the true thickness is more thinned compared to that provided by the “raw” or unprocessed image.

### 2.4. Statistical Analyses

Statistical analyses were conducted to compare the diagnostic performance and measurement values of original OCT-derived parameters versus those corrected using the AI-based compensation method. Descriptive statistics were calculated for each sector. Baseline characteristics were performed by two-sample *t*-tests and Chi-squared tests.

Normality was assessed using the Shapiro–Wilk test. Given the differences in variance and sample size between groups, Welch’s unequal variances *t*-test was used to compare mean thickness values across sectors between uncorrected and corrected datasets. To evaluate diagnostic performance, receiver operating characteristic (ROC) curves were constructed for each OCT parameter (both uncorrected and corrected), considering the presence of glaucoma as the reference standard. The area under the ROC curve (AUC) was calculated to quantify the discriminative ability of each parameter. Comparisons between AUCs of uncorrected and corrected measurements were performed using the DeLong test. The Pearson correlation coefficient was used to analyze the relationship between uncorrected and corrected RNFL thickness values in relation to the mean visual field defect. A *p*-value < 0.05 was considered statistically significant. Statistical analysis was performed using SPSS Statistics software, version 29.0 (IBM Corp., Armonk, NY, USA).

## 3. Results

Table 1 summarizes the baseline characteristics of each group. A total of 57 highly myopic eyes were included in the analysis, 31 from patients with glaucoma and 26 from non-glaucomatous individuals. The proportion of male participants was 55% in the glaucoma group and 54% in the control group (*p* = 0.94). The mean age was higher in the glaucoma group (64.1 ± 11.5 years) compared to the control group (58.3 ± 18.3 years), although this difference did not reach statistical significance (*p* = 0.17). Axial length (AL) was slightly greater in glaucomatous eyes (28.0 ± 1.4 mm) than in control eyes (27.5 ± 1.5 mm), without statistically significant differences (*p* = 0.63). Comparatively, the spherical equivalent was −8.71 ± 2.86 diopters in the control group and −9.10 ± 3.39 diopters in the glaucoma group (*p* = 0.64). Central corneal thickness (CCT) was similar in the control group (546.6 ± 18.7) compared to the glaucoma group (551.8 ± 17.5) (*p* = 0.14). A marked and statistically significant difference was observed in mean defect (MD) in the visual field (1.92 ± 1.22 dB in the control group vs. 6.83 ± 3.97 dB in the glaucoma group; *p* < 0.001). Similarly, best-corrected visual acuity measured using the ETDRS scale showed statistically significant differences (*p* < 0.001), with values of 82.69 ± 4.30 in the control group and 71.94 ± 12.76 in the glaucoma group. Intraocular pressure (IOP) was comparable between groups (16.35 ± 1.98 mmHg in controls vs. 15.81 ± 2.61 mmHg in the glaucoma group; *p* = 0.38). The number of hypotensive medications was 0.00 ± 0.00 in the control group and 1.74 ± 0.68 in the glaucoma group, showing a statistically significant difference (*p* < 0.001). Regarding prior surgeries, the following categories were identified: None (N), observed in 9 eyes (34.6%) in the control group and 15 eyes (48.4%) in the glaucoma group; and uncomplicated phacoemulsification with intraocular lens implantation (P), present in 17 eyes (65.4%) in the control group and 16 eyes (51.6%) in the glaucoma group. No statistically significant differences were found in the distribution of prior surgeries between groups (*p* = 0.46). Notably, no eyes had undergone glaucoma surgery.

Table 2 summarizes the mean thickness values of the circumpapillary RNFL and related sectors obtained from AI-corrected and uncorrected (original) measurements, stratified by glaucoma status. In glaucomatous eyes, AI-corrected values revealed statistically significant thinning in the global RNFL (G: 48.1 ± 11.6 µm) compared to non-glaucomatous eyes (54.5 ± 12.0 µm, *p* = 0.049). Among sectors, significant differences were observed in the NI sector for corrected values (*p* = 0.037), while no uncorrected sector reached statistical significance. In non-glaucomatous eyes, uncorrected measures were systematically higher and more variable, with the global RNFL averaging 88.5 ± 15.5 µm compared to 78.9 ± 19.5 µm in the glaucoma group. However, these differences did not translate into statistically significant contrasts for individual sectors.

ROC curves were constructed for each corrected and uncorrected RNFL parameter. The AUC values are presented in Table 3.

**Figure 2:**
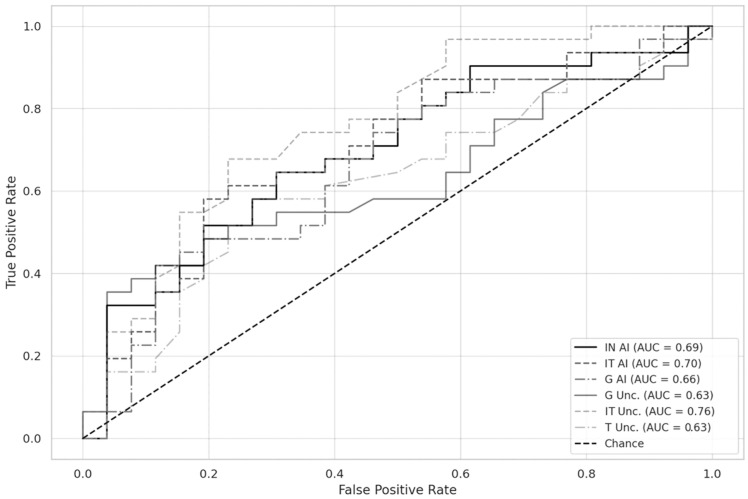
Receiver operating characteristic (ROC) curves for the three sectors with the highest diagnostic performance in both AI-corrected and uncorrected RNFL measurements. The inferior nasal (IN), inferior temporal (IT), and global (G) sectors showed the highest AUCs among corrected parameters, while inferior temporal (IT), global (G), and temporal (T) were the top performers in uncorrected measurements.

The best individual-performing corrected sector was the nasal inferior (NI AI Corrected, AUC = 0.69), followed by the global RNFL (G AI Corrected, AUC = 0.66). In contrast, their uncorrected counterparts demonstrated lower AUCs (G Uncorrected: 0.63; NI Uncorrected: 0.60). Although none of the pairwise comparisons between corrected and uncorrected AUCs reached statistical significance (all *p* > 0.05, DeLong test), a trend toward improved performance was observed in the AI-corrected values across most sectors (Figure 2).

A multivariable logistic regression model was developed to assess whether combining sectors could enhance diagnostic power. Forward stepwise selection identified an optimal AI-corrected model including the superior temporal (ST), superior nasal (SN) and inferior nasal (IN) sectors:

logit(*p*) = 5.560 − 0.062 × ST_AI + 0.057 × SN_AI − 0.094 × IN_AI

AUC = 0.797 (95% CI: 0.66–0.93); likelihood-ratio test χ^2^ = 13.79 (3 df), *p* = 0.003; AUC vs. 0.5 (DeLong) *p* < 0.001.

Table 4 shows the correlation between RNFL values by sector, comparing the AI-corrected data with the original, when these measurements are correlated with perimetric values. No statistically significant differences were found.

**Figure 3:**
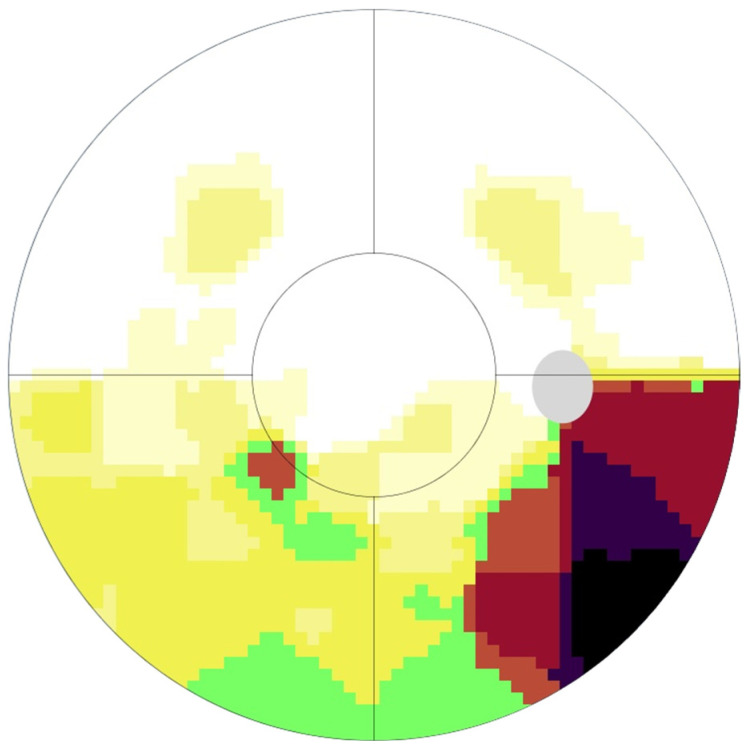
24-2 visual field of a patient with high axial myopia, in whom the perimetric defects are not consistent with the measurements obtained from the original OCT, but are concordant with the sectoral RNFL thinning identified by our tool. White areas indicate normal or near-normal retinal sensitivity. Yellow areas represent a mild reduction in sensitivity, whereas green areas suggest a moderate decrease. Red regions correspond to significant visual field defects, indicating markedly reduced sensitivity. Dark purple areas are associated with severe visual field loss, and black regions represent extremely severe loss or a complete absence of detectable visual sensitivity.

This 24-2 visual field from a 70-year-old patient included in our analysis, with an axial length of 26.88 mm and a mean deviation (MD) of −7.5 dB, demonstrates a typical glaucomatous defect characterized by an inferior arcuate scotoma with features consistent with a Bjerrum scotoma in his right eye. The defect shows a predominantly temporal distribution with moderate, incipient nasal involvement. Baseline, uncorrected peripapillary RNFL thickness values were 30 µm in the SN sector and 89 µm in the ST sector. After correction, these values decreased to 23.71 µm in SN and 42.84 µm in ST, corresponding to reductions of −20.97% and −51.87%, respectively. While the SN sector exhibited marked thinning that was already identified as outside normal limits on the original OCT, the ST sector was initially classified as within normal limits based on the uncorrected value (89 µm). In contrast, our AI-based correction model reclassified this sector as abnormal, with a substantially reduced thickness of 42.84 µm. In this case, the structural changes detected by our model—overlooked in the uncorrected imaging—demonstrate clear correspondence with the visual field defect. This improved structure–function agreement may support more informed clinical decision-making in the management of this patient (Figure 3).

## 4. Discussion

The differential diagnosis between myopic optic neuropathy (MON) and myopic glaucoma should be emphasized. The term MON refers to both the structural and functional alterations resulting from the anatomical changes observed in eyes with increased axial length characteristic of high myopia. As a general rule, the visual field defects associated with this entity are not characteristic of glaucomatous disease, but rather are specific to myopia. These include enlargement of the blind spot, temporal scotomas, and/or alterations secondary to peripapillary atrophy and/or staphylomas. Such visual field defects generally remain stable during longitudinal follow-up or exhibit only minimal changes. MON is usually considered to be independent of IOP, although elevated IOP may be present. Furthermore, MON may occur in young highly myopic patients, with a mean age typically lower than that observed in myopic glaucoma. By contrast, the key to the diagnosis of myopic glaucoma lies in the presence of typical glaucomatous visual field defects, as well as the detection of functional progression. This observation highlights the importance of obtaining several reliable and repeated visual field examinations over time in this group of patients. Patients with myopic glaucoma frequently present with elevated IOP levels or IOP values exceeding the individual tolerance threshold beyond which glaucomatous damage develops. Nevertheless, both entities may coexist, complicating the clinical management of these patients [11,12]. Building upon the aforementioned principles, our AI-based model could substantially facilitate the differential diagnosis between these two entities. First, the model operates by initially identifying the retinal layers and subsequently verifying both their continuity and segmentation integrity. Through this process, it is capable of recognizing the severe anatomical distortions that are characteristic of MON, thereby alerting the clinician to the presence of these highly atypical structural alterations. In addition, during longitudinal follow-up, the model would be expected to provide relatively stable measurements over time in patients affected by MON, consistent with the generally non-progressive nature of this condition. In contrast, when considering myopic glaucoma—a dynamic pathological entity with potential for progression—the system could provide clinicians with quantitative sectorial RNFL measurements that progressively reflect retinal nerve fiber layer thinning over time. Consequently, these longitudinal AI-derived metrics may serve as a valuable complementary diagnostic tool, offering additional clinical value in distinguishing stable myopic structural abnormalities from true glaucomatous progression.

Glaucoma specialists established the diagnosis of glaucoma in all cases. To this end, visual field characteristics (including characteristic visual field defects, mean deviation, and progression), baseline IOP with and without treatment, as well as patient age and optic nerve head characteristics, were evaluated, as detailed in Section 3.

Finally, our AI module verifies the continuity and correct segmentation of retinal layers in the peripapillary region as preliminary steps before performing thickness correction. As described in Section 2, cases with poor image quality were excluded, thereby ensuring that none of the patients included in our sample presented the extreme posterior segment deformities typically associated with MON.

In this study, we evaluated the diagnostic utility of AI-corrected circumpapillary RNFL thickness values in differentiating glaucomatous from non-glaucomatous eyes in a cohort of highly myopic patients. The application of deep learning-based segmentation correction significantly enhanced diagnostic accuracy, particularly in the IN, IT and global RNFL sectors. Our results underscore the limitations of standard uncorrected RNFL analysis in this population and demonstrate the clinical value of AI-enhanced structural biomarkers for glaucoma detection.

Regarding baseline characteristics, no notable differences were observed in sex distribution across groups (53.8% male in the control group and 54.8% male in the glaucoma group). Similarly, no statistically significant differences were found in age, although there was a tendency toward a slightly higher mean age in the glaucoma group (64.1 years vs. 58.3 years in the control group), which is consistent with the literature, as advanced age is a known risk factor for glaucoma [7]. Likewise, the mean axial length was somewhat greater in the glaucoma group (28.00 mm vs. 27.50 mm in the control group), which is also in line with existing evidence, considering that increased axial length is associated with a higher prevalence of myopic glaucoma [7]. Consistent with axial length, spherical equivalent (SE) was also slightly higher in the glaucoma group (−9.10 D) compared to the control group (−8.71 D). CCT didn’t display any differences between groups (546.6 µm in the ‘no glaucoma’ group vs. 551.8 µm in the glaucoma group). Best-corrected visual acuity (VA) showed statistically significant differences, being higher in the control group than in the glaucoma group (82.69 ETDRS letters vs. 71.94 ETDRS letters). Beyond glaucomatous visual field damage, this difference may also be related to the higher frequency of previous ocular surgeries in the control group (65.4%) compared to the glaucoma group (51.6%). IOP did not differ significantly between groups (16.35 mmHg in the control group vs. 15.81 mmHg in the glaucoma group), likely reflecting differences in the use of hypotensive medications (none in the control group vs. a mean of 1.74 medications in the glaucoma group).

The global RNFL thickness was significantly lower in glaucomatous eyes after AI correction (48.1 ± 11.6 µm) compared to non-glaucomatous controls (54.5 ± 12.0 µm; *p* = 0.049). Additionally, the IN sector showed significant thinning in glaucoma (*p* = 0.037), while no sector reached significance in the uncorrected analysis. This result suggests that segmentation distortions in uncorrected OCT data may mask real structural damage, an effect previously described in highly myopic anatomy where disc tilt, peripapillary atrophy, and axial elongation alter RNFL topography and reduce reliability of standard segmentation algorithms [13].

ROC analysis further supported the benefit of AI correction. The highest AUC among corrected values was found in the nasal inferior sector (0.69), followed by the global RNFL (0.66). Although these values did not reach statistical significance compared to their uncorrected counterparts (IN: 0.60; G: 0.63), the consistent improvement across most sectors suggests that AI segmentation compensates for geometrical distortions and improves diagnostic robustness. This aligns with the findings of Poon et al., who showed that OCT segmentation artifacts are present in over 50% of highly myopic eyes, particularly in inferior and nasal sectors, and can mislead clinical interpretation if not corrected [14].

Furthermore, the benefit of combining multiple AI-corrected RNFL sectors was demonstrated in our multivariable model, which included the superior temporal (ST), superior nasal (SN) and inferior nasal (IN) sectors and achieved an AUC of 0.797 (*p* = 0.003). This performance is notably higher than that of the best individual sector (IN, AUC = 0.69), indicating a synergistic effect when spatially complementary sectors are combined. The selection of the IN sector is consistent with the well-established vulnerability of the inferior arcuate bundles in early glaucomatous damage, while the inclusion of ST and SN reflects the value of integrating information from the superior hemifield, where preperimetric defects in myopic glaucoma are also frequently observed. Jiang et al. showed that temporal RNFL thinning is particularly accelerated in highly myopic patients with open angle glaucoma, and that damage in this region may be underrepresented by traditional circular scan metrics [15].

Interestingly, uncorrected RNFL values in non-glaucomatous eyes were systematically higher and more variable (e.g., G: 88.5 ± 15.5 µm) compared to their corrected counterparts, likely due to inclusion of peripapillary structures and misidentification of RNFL boundaries. This phenomenon, sometimes referred to as “false thickening,” has been widely documented and contributes to diagnostic uncertainty in high myopia [16]. Our findings show that AI correction helps normalize these discrepancies and allows better separation between glaucomatous and non-glaucomatous eyes, thereby reducing both false negatives and false positives.

The improved performance of AI-corrected models is consistent with results obtained using alternative metrics such as BMO-MRW. Kudsieh et al. reported that BMO-MRW achieves greater specificity and agreement with clinical classification than RNFL in highly myopic populations, due to its independence from disc tilt and peripapillary atrophy [17]. While BMO-MRW was not evaluated in our study, the AI-corrected RNFL measurements we employed may functionally approximate similar correction by eliminating segmentation-related biases.

Other studies have emphasized the importance of macular parameters, such as ganglion cell complex (GCC) or GCIPL thickness, in high myopia. Wang et al. demonstrated that macular thickness metrics outperformed RNFL for glaucoma detection in high myopia (GCC AUC: 0.968 vs. RNFL AUC: 0.855) [18]. Although our analysis was limited to RNFL, the strong performance of the multivariable model (AUC: 0.79) suggests that regional RNFL information, when properly corrected, may complement macular metrics in predictive value, especially in cases presenting with other concomitant macular diseases, a circumstance particularly common in populations with high axial myopia.

Moreover, our findings are relevant in the context of clinical workflow, where the use of uncorrected RNFL measurements frequently leads to “red disease” false positives due to normal variations in myopic anatomy not represented in the normative database [19]. The application of AI correction, particularly one trained on high myopia-specific datasets, offers a clinically feasible solution to this long-standing problem, allowing more accurate risk stratification and minimizing unnecessary interventions.

Thinning of RNFL frequently precedes glaucomatous visual field defects, serving as an indicator of the early stages of glaucoma [20]. Furthermore, the numerical indices derived from visual field analysis, such as mean deviation (MD) or visual field index (VFI), can hardly be directly and unequivocally correlated with retinal nerve fiber layer (RNFL) thickness as measured by optical coherence tomography (OCT), whether using raw values or those corrected by artificial intelligence, as in the case of our model. It is important to consider the presence of generalized depressions due to conditions such as cataract, as well as visual field defects inherent to high myopia (e.g., enlarged blind spot, vertical step, partial peripheral rim) [21], or even those secondary to chorioretinal atrophy, which is undeniably associated with axial myopia [22].

Lastly, these results support recent calls to integrate AI-enhanced OCT imaging into routine clinical evaluation of glaucoma in myopic populations. With further development, AI tools could be expanded to include macular segmentation, BMO-MRW estimation, and even vascular imaging from wide-field OCT angiography (WF-OCTA), which has shown comparable sensitivity and superior specificity for detecting HMG compared to standard RNFL imaging [23,24].

The principal strengths of our tool lie in its applicability to a population with very high axial myopia—frequently excluded from previous studies—as well as in the composition of our sample, which includes an older cohort and is therefore more representative of real-world clinical practice.

### Limitations

The primary strength of this study is its potential to contribute to the diagnosis of complex cases. At the same time, it is important to acknowledge that a variable proportion of highly myopic eyes cannot be reliably evaluated using RNFL OCT, owing to their intrinsic extreme cytoarchitectural alterations. Nonetheless, we consider that enhancing diagnostic accuracy in a significant subset of high myopia patients remains clinically meaningful, and that the potential benefits offered by this approach support its relevance notwithstanding the aforementioned. There are other limitations of a different nature that should be mentioned. Regarding the methodology of this AI tool, it is important to recall how it operates: it corrects image tilt based on the posterior curvature secondary to increased axial length. This does not exempt the tool from perpetuating other errors in retinal layer segmentation, such as the inclusion of peripapillary structures, a condition that may happen in highly axial myopia. As previously explained, the images provided serve merely as a visual aid and are not part of the working algorithm, which could imply the absence of a final verification step allowing the clinician to confirm that the correction process yields plausible results. Finally, when considering its applicability, its main limitation lies in the lack of integration of this technology into the widely used OCT platforms currently available.

## 5. Conclusions

In summary, AI-corrected RNFL thickness values significantly enhance the diagnostic capacity of OCT for glaucoma detection in highly myopic eyes. Statistically significant thinning in global and nasal inferior sectors, along with improved AUC values across most parameters and a multivariable model with an AUC of 0.79, underscore the potential clinical benefit of AI segmentation. These results align with recent evidence on the limitations of conventional OCT in high myopia and provide further justification for integrating AI-based correction into clinical practice.

## Figures and Tables

**Table 1:** Patient and eye characteristics by diagnosis. M = Male; F = Female; AL = Axial Length; SE = Spherical Equivalent; CCT = Central Corneal Thickness; MD = Mean Deviation; VA = Visual Acuity; IOP = Intraocular Pressure; N = None; P = Phacoemulsification + IOL. Continuous variables are presented as mean ± standard deviation. Statistical comparisons were performed by two-sample *t*-tests and Chi-squared tests.

Variable	No Glaucoma	Glaucoma	*p*-Value
n	26 (45.6%)	31 (54.4%)	*p* = 0.51
Gender	M: 14 (53.8%) F: 12 (46.2%)	M: 17 (54.8%) F: 14 (45.2%)	*p* = 0.94
Age (years)	58.3 ± 18.3 years	64.1 ± 11.5 years	*p* = 0.17
AL (mm)	27.5 ± 1.5 mm	28.0 ± 1.4 mm	*p* = 0.63
SE (diopters)	−8.71 ± 2.86	−9.10 ± 3.39	*p* = 0.64
CCT (µm)	546.6 ± 18.7	551.8 ± 17.5	*p* = 0.14
MD (dB)	1.92 ± 1.22	6.83 ± 3.97	*p* < 0.001
VA (ETDRS)	82.69 ± 4.30	71.94 ± 12.76	*p* < 0.001
IOP (mmHg)	16.35 ± 1.98	15.81 ± 2.61	*p* = 0.38
Treatment (nº of drugs)	0.00 ± 0.00	1.74 ± 0.68	*p* < 0.001
Surgery	N: 9 (34.6%) P: 17 (65.4%)	N: 15 (48.4%) P: 16 (51.6%)	*p* = 0.44

**Table 2:** Comparison of AI-corrected and uncorrected peripapillary RNFL thickness values in glaucomatous and non-glaucomatous eyes, stratified by sector. Values are presented as mean ± standard deviation (µm). Statistical comparisons between corrected and uncorrected values within each group were performed using Welch’s *t*-test.

Sector	AI-Corrected (Glaucoma)	Uncorrected (Glaucoma)	*p*-Value	AI-Corrected (No Glaucoma)	Uncorrected (No Glaucoma)	*p*-Value
Global	48.10 ± 11.60	78.90 ± 15.50	*p* < 0.001	54.50 ± 12.00	88.50 ± 16.00	*p* < 0.001
ST	53.90 ± 12.30	69.00 ± 14.80	*p* < 0.001	61.90 ± 12.50	76.30 ± 15.30	*p* < 0.001
SN	46.00 ± 11.10	61.50 ± 14.00	*p* < 0.001	46.70 ± 11.20	67.00 ± 14.20	*p* < 0.001
N	43.00 ± 10.80	60.00 ± 13.70	*p* < 0.001	48.50 ± 11.00	67.80 ± 14.00	*p* < 0.001
IN	44.30 ± 11.00	65.40 ± 15.20	*p* < 0.001	51.30 ± 11.40	71.20 ± 15.50	*p* < 0.001
T	55.00 ± 11.50	70.00 ± 14.60	*p* < 0.001	59.20 ± 11.70	75.60 ± 15.00	*p* < 0.001
IT	61.60 ± 12.00	76.80 ± 15.00	*p* < 0.001	66.20 ± 12.10	81.30 ± 15.40	*p* < 0.001

**Table 3:** Area under the receiver operating characteristic curve (AUC) values for each RNFL sector, comparing AI-corrected and uncorrected measurements for glaucoma detection. *p*-values indicate statistical comparison of AUCs between corrected and uncorrected parameters using the DeLong test.

Sector	AI-Corrected AUC	Uncorrected AUC	*p*-Value
Global	0.66	0.63	0.170
ST	0.62	0.52	0.210
SN	0.52	0.46	0.440
N	0.61	0.54	0.400
IN	0.69	0.60	0.180
T	0.64	0.59	0.250
IT	0.67	0.63	0.220

**Table 4:** Pearson correlation (r) between AI-corrected and uncorrected sectors in the glaucoma patient group, when correlating OCT thickness measurements with MD values from the visual field. *p*-values indicate the statistical comparison of correlation coefficients between corrected and uncorrected parameters.

Sector	AI-Corrected r (Pearson)	Uncorrected r (Pearson)	*p*-Value
Global	−0.672	−0.623	*p* = 0.58
ST	−0.597	−0.671	*p* = 0.41
SN	−0.452	−0.371	*p* = 0.49
N	−0.402	−0.261	*p* = 0.28
IN	−0.257	−0.471	*p* = 0.11
T	−0.255	−0.445	*p* = 0.21
IT	−0.318	−0.414	*p* = 0.33

## Data Availability

The datasets generated and analysed during the current study that support the results are available from the corresponding author on reasonable request, due to privacy reasons.

## Abbreviations

The following abbreviations are used in this manuscript:

OCT	Optical Coherence Tomography
RNFL	Retinal Nerve Fiber Layer
AI	Artificial Intelligence
MGU-Net	Multiscale Gate Attention Network
ST	Superior Temporal
SN	Superior Nasal
N	Nasal
IN	Inferior Nasal
T	Temporal
IT	Inferior Temporal
ROC	Receiver Operating Characteristic
AUC	Area Under the Curve
BMO-MRW	Bruch Membrane Opening—Minimum Rim Width
GCIPL	Ganglion Cell Inner Plexiform Layer
AUROC	Area Under the Receiver Operating Characteristic Curve
3D-NRR	Three-Dimensional Neuroretinal Rim Thickness
GCC	Ganglion Cell Complex
WF-OCTA	Wide Fundus-Optical Coherence Tomography Angiography
AL	Axial Length
SE	Spherical Equivalent
CCT	Central Corneal Thickness
MD	Mean Deviation
VA	Visual Acuity
ETDRS	Early Treatment Diabetic Retinopathy Study
IOP	Intraocular Pressure
MON	Myopic Optic Neuropathy
